# Developing and Applying a Single Strategy for Improved Intestinal Permeability of Diverse and Complex Phytomolecules: Nanoformulations of Rutin, Quercetin, Thymoquinone Provide Proof-of-Concept

**DOI:** 10.34172/apb.39294

**Published:** 2024-09-15

**Authors:** Rajani Mathur, Sahiba Khan, Ruchi Tripathi, Saima Amin, Saumitra Dey Choudhary

**Affiliations:** ^1^Department of Pharmacology, Delhi Institute of Pharmaceutical Sciences & Research, DPSRU, New Delhi, India; ^2^Pharmaceutics School of Pharmaceutical Education & Research, Jamia Hamdard, New Delhi, India; ^3^All India Institute of Medical Sciences, New Delhi, India

**Keywords:** Rutin, Quercetin, Thymoquinone, Eudragit, Nanoparticle, Apparent permeability

## Abstract

**Purpose:**

The clinical use and efficacy of phytomolecules are often hampered as their complex structure, poor aqueous solubility and low biological stability restricts their intestinal permeability which results in low oral bioavailability. Rutin (RT), quercetin (QU), thymoquinone (TQ) are few of such potent and therapeutically versatile phytomolecules that await maximal utilization. To address this lacuna, an attempt was made to develop a single strategy for enhanced intestinal permeation that can be applied to diverse phytomolecules.

**Methods:**

A simple idea with easy-to-apply method was developed that involved preparing nanoparticles of the phytomolecules RT, QU, TQ using Eudragit matrix (RT-PNP, QU-PNP, TQ-PNP) and examined for particle characteristics, EE, in vitro release and kinetics. Phytomolecule loaded nanoparticle (PNPs) were encapsulated in HPMC grade capsule shell and evaluated for intestinal permeability by everted gut sac method.

**Results:**

The average particle sizes of RT-PNP, QU-PNP, TQ-PNP were 446±0.152, 39.6±0.006 and 186±0.513 nm, polydispersity indices were<0.5 with negative zeta potential. The % release of respective phytomolecule from RT-PNP, QU-PNP, TQ-PNP was significantly higher (*P*<0.05) at pH 6.8 than pH 1.2. PNPs followed Higuchi kinetics with non-Fickian diffusion mechanisms. The apparent intestinal permeability (Papp) of RT-PNP, QU-PNP, TQ-PNP were 14.45±4.85, 12.96±1.73 and 30.87±8.75 µg/cm^2^, respectively, significantly (<0.5) greater vs RT, QU, TQ, respectively. CLSM confirmed significantly higher (*P*<0.05) intestinal permeation of RT-PNP, QU-PNP, TQ-PNP vs RT, QU, TQ, respectively.

**Conclusion:**

Developed PNPs appear to be a good approach to increase the permeability of hydrophobic phytomolecules.

## Introduction

 Formulations containing phytomolecules have flooded the market as they promise effective and safe treatment and prevention of various pathological states particularly, lifestyle diseases that require lifelong medication. Despite the wide-acceptability and usage of phytomolecules, their clinical advantage remains underutilized largely due to their complex structure, poor solubility, and instability in the biological milieu that affects their bioavailability and therapeutic advantage.

 Rutin (RT, 2-(3,4-dihydroxyphenyl)-4,5-dihydroxy-3-[3,4,5-trihydroxy-6-[(3,4,5-trihydroxy 6methyl-oxan-2-yl)oxymethyl]oxan-2-yl]oxy-chromen-7-one) and quercetin (QU, 3,3′,4′,5,7‐pentahydroxyflavone) are polyphenolic flavonoid compounds that are widely distributed in plants and have wide therapeutic applications due to their quenching property.^1-3^ Another phytomolecule, Thymoquinone (TQ, 2-isopropyl-5-methyl-1, 4-benzoquinone), is the main active constituent of the volatile oil, obtained from the seeds of *Nigella sativa* (Ranunculaceae) and holds tremendous potential as a therapeutic phytomolecule.^4^

 Traditionally, the oral route of drug administration for pharmacologically active compounds is preferred, as it holds many advantages like patient compliance, ease of use, safe, cost-effective, etc. In cases of RT, QU, TQ, their pharmacological utilization following oral administration is restricted due to poor solubility and dissolution at physiological pH and poor absorption and bioavailability.^5,6^ One of the reasons behind low oral bioavailability of complexly structured active molecules is low permeation across intestinal epithelium, a critical rate-limiting step.^7^

 Active research is being conducted to develop strategies to enhance the intestinal permeability of active molecules, one of which is the development of nanoparticle-based formulation.^8,9^ Oral polymeric nanoparticles have attracted considerable attention as novel drug delivery carriers as they are implicated to enhance the bioavailability, stability and efficacy of incorporated drugs.^10^ As compared to colloidal carriers, the polymeric nanoparticles are resistant to degradation in the gastrointestinal tract and can protect encapsulated drugs from the gastrointestinal environment.

 Therefore, development of polymeric nano sized delivery system is one of the most promising areas for oral drug delivery of the phytomolecules as it can be adopted to provide targeted delivery, with improved aqueous solubility of complex phytomolecules. Eudragit based polymer matrix are commonly used for enteric coating and also for preparation of controlled-release dosage forms.^11^ Eudragit RL100 and Eudragit RS100 are copolymers of ethyl acrylate, methyl methacrylate, and a low content of a methacrylic acid ester with quaternary ammonium groups. The ammonium groups are present as salts and make the polymers permeable across the cell membrane following drug delivery.^12,13^

 In the present study, a novel strategy has been adopted and applied to prepare and optimize nanoformulations of RT, QU and TQ with improved intestinal permeability.

## Materials and Methods

###  Chemicals 

 RT and QU were purchased from Loba Chemie PVT. Ltd, India. TQ (purity > 98%) and polyvinyl alcohol (PVA) were purchased from Sigma-Aldrich, Germany.

###  Optimization parameters 

####  Calibration curve

 The standard stock solution (0.1 mg/mL) of RT was prepared in methanol while those of QU, TQ were prepared in ethanol. The standard stock solutions were serially diluted (2–20 μg/ mL) in respective solvents and the concentration versus absorbance calibration curves of RT, QU, TQ were measured at 268, 374, 260 nm, respectively (Thermo Fisher Scientific, AquaMate 800, USA).

 For the purpose of, in vitro release the standard stock solution of RT, QT, TQ were prepared in phosphate buffer (pH 6.8), serially diluted (2–20 μg/mL) and plotted at 268, 374, 260 nm, respectively.

####  Solubility 

 The solubility of RT, QU, TQ were determined in different solvents (n-octanol, methanol, ethanol, dichloromethane (DCM) and water) by the shake flask method. Briefly, surplus amounts of RT, QU, TQ were added to each of the solvent (5 mL), vortexed (15 minutes), continuously mixed (37 °C, 24 hours), filtered (0.45μm) and the amount of dissolved phytomolecule was determined from the calibration curves as at Section 2.2.1.^14^

####  Partition coefficient 

 The partition coefficients (log p) of RT, QU, TQ were determined as ratio of concentration of the respective phytomolecule in n-octanol to concentration of the phytomolecule in water at 37 ± 0.5 °C using the calibration curves as at Section 2.2.1.^15,16^

####  Effect of polymer concentration on percentage yield, drug entrapment efficiency and percentage drug loading 

 For the preparation of phytomolecule loaded nanoparticles (PNPs) of RT, QU, TQ, and the respective phytomolecules were added to the polymers in a fixed ratio (1:5). For the purpose of optimization, the polymers Eudragit RS100 and Eudragit RL100 were used in three different ratios (30:70, 50:50, 70:30), while keeping the amount of stabilizer constant (PVA, 0.25% w/v) so that three batches of PNPs of each phytomolecule, namely, RT-PNP (1A, 1B, 1C), QU-PNP (2A, 2B, 2C), TQ-PNP (3A, 3B, 3C) were prepared.

###  Preparation of phytomolecule loaded nanoparticles and formulation development

 In accordance with the reported solvent evaporation method, the RT-PNP, QU-PNP, TQ-PNP were prepared and optimized as detailed in Section 2.2.4.^17,18^ Briefly, RT, QU, TQ were dissolved in methanol, ethanol, DCM (1:1), respectively. The optimized polymer ratio (Eudragit RS100: RL100: 30:70) was dissolved in ethanol (5 mL) with stirring (30 minutes). The organic phase was prepared by mixing dissolved phytomolecule with polymer solution. The aqueous phase was prepared by mixing double distilled water (50 mL) with PV (0.25%). Next, the organic phase was added dropwise into the aqueous phase with continuous stirring (2 hours, 40 °C). The resultant dispersion was sonicated, frozen (−20 °C, 12 hours) and lyophilized. Finally, lyophilized freeze dried powder of RT-PNP, QU-PNP, TQ-PNP were filled in HPMC grade capsule shell and marked as RT-For, QU-For, TQ-For, respectively. Blank formulations were also prepared using the same procedure sans RT, QU, TQ.

 The optimized batches of RT-PNP, QU-PNP, TQ-PNP were deduced from the effect of polymer concentration on percentage yield, entrapment efficiencies and percentage drug loading.

###  Characterization of phytomolecule loaded nanoparticles

####  Determination of the percentage yield

 The freeze-dried RT-PNP, QU-PNP, TQ-PNP were collected and weighed accurately. The percentage yield was calculated as the ratio of the weight of PNPs to total weight of phytomolecules and polymers.^19^

####  Determination of percentage of entrapment efficiency and percentage drug loading 

 To determine the amount of phytomolecule entrapped in the PNPs, the RT-PNP, QU-PNP, TQ-PNP were separated from the dispersion containing free phytomolecule by centrifugation. Following solvent evaporation, the obtained dispersion was centrifuged (12 000 rpm, 30 minutes at 4 °C) and the amount of free phytomolecules (RT, QU, TQ) in the supernatant was measured using calibration curves as at Section 2.2.1. The amount of phytomolecule entrapped into PNP was calculated as the difference between the phytomolecule used for the formulation and the amount of phytomolecule in the supernatant. The entrapment efficiency (EE%) was calculated as the ratio of difference between total amount of phytomolecule added and free phytomolecule to the total amount of phytomolecule added.^20^ The weighed amount of final freeze dried RT-PNP, QU-PNP, TQ-PNP were dissolved into the acetone and analysed as detailed in Section 2.2.1

 The percentage phytomolecule loading was calculated by the formula:

 Phytomolecule loading = Amount of phytomolecule entrapped/(Amount of phytomolecule added) + (Amount of Excipients added)

####  Determination particle size, polydispersity index and zeta potential 

 The freeze dried RT-PNP, QU-PNP, TQ-PNP were resuspended separately, in distilled water and diluted up to 40 times. The obtained diluted suspensions were analyzed for particle size and polydispersity index (PDI) using dynamic light scattering (Zeta-Sizer, Malvern, Nano Series ZS90, Malvern Instruments, Ltd., UK). The zeta potential of RT-PNP, QU-PNP, TQ-PNP were determined by placing the diluted sample in elecrophoretic cell of the same instrument.^21^

####  Transmission electron microscopy

 The transmission electron microscopy **(**TEM) of RT-PNP, QU-PNP, TQ-PNP was performed by negative staining with phosphotungstic acid (PTA). A drop of dispersion, as prepared in Section 2.3 was placed on a carbon-coated copper grid and air-dried (10 minutes), followed by the addition of a drop of PTA (1% *w/v*) and left undisturbed (3 minutes). The excess liquid was dabbed before TEM imaging (TECNAI 200 Kv TEM).^22^

####  Differential scanning calorimetry analysis

 The compatibilities of RT, QU, TQ with polymers were analysed (Shimadzu DSC-60, Tokyo, Japan). To achieve this, 5 mg of RT, QU, TQ, Eudragit RS100, Eudragit RL100, RT-PNP, QU-PNP, TQ-PNP were placed in separate aluminium pans and crimped. The sealed pans were heated under nitrogen atmosphere (10 mL/min) from 25 °C to 300 °C at a heating rate of 10 °C/min. An empty aluminium pan was used as the reference pan.^23^

####  Nuclear magnetic resonance

 The RT, QU, TQ, Eudragit RS100, Eudragit RL100, RT-PNP, QU-PNP, TQ-PNP were dissolved in deutero chloroform (CDCl_3_) and subjected to ^1^H-NMR for structural analysis (Bruker 700 MHz Ultra Shield NMR).^24^

####  In vitro release study

 In accordance with the standard protocols, the *in vitro* release from RT-For, QU-For, TQ-For were conducted. Briefly, the RT-For, QU-For, TQ-For were placed in buffer solutions simulating gastric (10 mL, pH 1.2) and intestinal (10 mL, pH 6.8) environment for 6 hours under standard conditions (37 °C ± 0.5 °C, 100 rpm). At predetermined time intervals (0, 15, 30, 45, 60, 90, 120 and 180 minutes), sample aliquots (2 mL) were withdrawn and equal volumes of fresh buffer solution were replaced to ensure the sink conditions.

 The RT, QU, TQ (1 mg) were subjected to the same procedure and drug content was analyzed using calibration curves from Section 2.2.1. The cumulative release percentages were calculated as the ratio of the amount of phytomolecule released to the initial amount of phytomolecule in the capsule, at each time interval. The cumulative percentage of phytomolecule released versus time curves were plotted and the release efficiencies were calculated.^25^

 The following equation was used to calculate the cumulative percentage release:

 Concentration of phytomolecule (µg/mL) = (slope × absorbance) ± intercept

 Amount of phytomolecule release (mg/ mL) = Concentration of phytomolecule × Dissolution bath volume × dilution factor/1000

 Cumulative percentage = Volume of sample withdrawn (mL) × P (t – 1) + Pt release (%) / Bath volume (v)

 Where Pt = Percentage release at time t and P (t – 1) = Percentage release previous to ‘t’

####  Release kinetics

 In order to understand the release kinetics and mechanism, the results of *in vitro *release studies of RT-PNP, QU-PNP, TQ-PNP were fitted to various kinetic equations such as zero order (cumulative % release vs. time), first order (log % drug remaining vs. time), Higuchi’s model (cumulative % drug release vs. square root of time) and Korsmeyer–Peppas model (log of % drug release vs. log time) and R^2^ values were determined.^26,27^ In the Korsmeyer–Peppas model, the n value was applied to determine the release mechanism as described below:

 n < 0.5 (0.45)—quasi-Fickian diffusion,

 n = 0.5 (0.45)—diffusion mechanism,

 0.5 (0.45) < n < 1—non-Fickian diffusion,

 n = 1 (0.89)—case II transport (zero-order release),

 n > 1 (0.89)—super case II transport

###  Stability studies

 Stability studies were performed to evaluate the effect of storage conditions on the physicochemical parameters of RT-For, QU-For, TQ-For. The optimized RT-For, QU-For, TQ-For were stored in sealed glass vials (40 °C ± 2°C / 75% RH ± 5% RH) protected from light, for 3 months. The stored and freshly prepared RT-For, QU-For, TQ-For were evaluated for their physical appearance, entrapment efficiency (%), drug loading (%) and *in vitro* release profiles.

###  Ex-Vivo intestinal permeability study

####  Animals

 Adult Wistar male albino rats (200–250 g) were maintained under standard laboratory conditions, (25 ± 2 °C, 55 ± 5%) and provided normal chow and filtered drinking water, *ad libitum*. The study was performed according to the protocol approved by the standing Institutional Animal Ethics Committee (IAEC 2019/II 09).

####  Apparent permeability

 In accordance with the reported protocols, the apparent permeability, Papp, for RT-PNP, QU-PNP, TQ-PNP were determined.^28^ After overnight fasting, the rats were anesthetized (Ketamine: xylazine::87:13 mg/kg) and midline abdominal incisions were made to excise intestine. The intestine was cut into equal segments (10 cm), flushed with normal saline to clear the contents, and then immersed in ice-cold Krebs solution that was pregassed with carbogen. Each segment was inverted by gently pushing a smooth glass rod and then filled with 2mL of Krebs solution. Both ends of each segment were secured with a thread forming an everted gut sac. The distended gut sac was placed in 50mL of Krebs-Ringer solution containing RT-PNP, QU-PNP or TQ-PNP (1 mg), continually aerated with 5% CO_2_ and 95% O_2_ and maintained at 37 ± 0.5 °C. The sample aliquots (100 µL) were withdrawn from serosal solution at different time intervals (0, 5, 10, 15, 30, 60,120 and 160 minutes). The aliquots were assayed for the content of respective phytomolecule using calibration curves at Section 2.2.1. The same procedure was repeated with RT, QU, TQ (1 mg). The apparent permeability, Papp was calculated as dQ/dt x1/AC, where dQ/dt is the permeability rate, C_0_ is the initial concentration over the mucosal side and A is the surface area.

####  Validation of ex-vivo gut permeation study by confocal laser scanning microscopy using rhodamine B as fluorescent dye 

 Male albino wistar rats were anesthetized with Ketamine: xylazine::87:13 mg/kg, and 2 cm loops of ileum from the intestinal section were made and washed with Krebs–Ringer solution (37 °C). Rhodamine B dye solution (0.5mL) and Rhodamine B loaded PNPs with dilution (10 M) were filled into the loop, ligated at both ends and kept for incubation into the phosphate buffer saline (pH 6.8). After 1 hour, the section of the loop was removed and washed using Krebs–Ringer solution to remove excess amount of the dye and fixed.

 The extent of the penetration of Rhodamine B dye in the *z*-axis was analyzed by confocal laser scanning microscopy (CLSM, Zeiss LSM980).^29^ To calculate the fluorescence intensities, images were captured using a plan apochromat (40X, 0.95 NA objective). All fluorescence images were captured under identical settings for every experimental set. Images were captured at 1X and 2.5X optical zoom and the 2.5X images were used for quantification. Fluorescence intensities were calculated using ImageJ/Fiji software. All control and experimental tissue sections were processed in the same way. Maximum intensity projections (MIPs) were first generated followed by image thresholding. A binary mask was then created from the thresholded image and applied to the original image to extract the intensity density values from manually selected regions.

###  Statistical analysis

 The results of percentage yield, percentage entrapment efficiency, percentage drug loading, *in vitro* drug release, apparent permeability, were presented as the mean ± standard deviation (SD). Statistical analysis was performed with the unpaired Student’s *t* test to compare the means between two groups using the software Graph Pad Prism *ver*5.0 (San Diego, CA, USA). For CLSM, error bars in the histograms represent standard error of the mean (SEM). A value of *P* < 0.05 was considered as statistically significant. *P < 0.05 and ***P < 0.001.

## Results and Discussion

###  Calibration Curves of rutin, quercetin, thymoquinone 

 The standard curves of concentration versus absorbance of RT, QU, TQ showed a linear relationship and R^2^ were 0.953, 0.945, 0.924, respectively. The R^2^ value of RT, QU, TQ in phosphate buffer (pH 6.8) were 0.953, 0.945, 0.924, respectively.

###  Solubility of rutin, quercetin and thymoquinone

 The aqueous solubility of RT, QU, TQ were 0.2, 0.1, 0.2 mg/mL respectively. The ethanol solubility of RT, QU, TQ were 0.5, 0.7, 0.9 mg/mL, respectively. The solubility of RT,QU,TQ were higher in ethanol than water, that make a case for adopting pharmaceutical approaches to enhance their aqueous solubility, as desirable following oral administration.

###  Partition coefficient

 The partition coefficients (P) of RT, QU, TQ were 0.69, 0.35, 0.30, respectively indicating that they are sparingly hydrophilic in nature. As the partition coefficient is an important criteria that determines the partitioning of the molecules across the membranes in the body, a pharmaceutical approach may be adopted to circumvent the associated challenges of poor absorption and permeability.

###  Optimization of ratio of Eudragit RL100 and Eudragit RS100 for polymeric matrix

 The phytomolecule-polymer concentration was fixed at 1:5 in the organic phase, while PVA was constant at 0.25% in the aqueous phase. The eﬀects of different ratios of Eudragit RL100 and Eudragit RS100 on entrapment efficiency (EE %), drug loading (DL %) and yield (%) are tabulated (Table 1). The EE% and yield% were decreased when the ratio of the Eudragit RS100: Eudragit RL100 was less than 70:30.

**Table 1:** Composition of phytomolecules loaded PNPs batches

**Phytomolecules**	**PNPs Batch no.**	**Polymer combination (RS:RL)**	**Solvents (5mL)**	**Drug polymer ratio**	**Stabilizing agent (PVA)**
RT	Batch 1-A	30:70	Methanol	1:5	0.25%
	Batch 1-B	70:30	Methanol	1:5	0.25%
	Batch 1-C	50:50	Methanol	1:5	0.25%
QT	Batch 2-A	30:70	Ethanol	1:5	0.25%
	Batch 2-B	70:30	Ethanol	1:5	0.25%
	Batch 2-C	50:50	Ethanol	1:5	0.25%
TQ	Batch 3-A	30:70	DCM	1:5	0.25%
	Batch 3-B	70:30	DCM	1:5	0.25%
	Batch 3-C	50:50	DCM	1:5	0.25%

 Eudragit RS has fewer quaternary ammonium groups (4.5 to 6.8%) than Eudragit RL (8.8 to 12%), which makes the latter hydrophilic. The availability of quaternary ammonium groups in Eudragit RL100, as opposed to Eudragit RS100, may have raised the EE% when the concentration of former was increased from 30% to 70%.^30^

###  Optimization of PNP batches from percentage yield, encapsulation efficiency and drug loading

 The % yield, EE% and DL% are important factors for optimizing nano-based carriers.^31^ The % yield, EE% and DL% of 3 batches each of RT-PNP, QU-PNP, TQ-PNP are tabulated in Table 2. The % yield of batches 1-A, 2-A and 3-B were 87, 91.02 and 75% respectively which was highest among all the prepared respective PNP-batches. The EE% of batches 1-A, 2-A and 3-B was 76.32, 73.28 and 60.76 % respectively (Table 2). The DL% of batches 1-A, 2-A and 3-B was 69.38, 66.61 and 55.24% respectively (Table 2). Based on these results, the batches 1-A, 2-A, 3-B of RT-PNP, QT-PNP, TQ-PNP, respectively were optimized and selected for further studies.

**Table 2:** EE%, DL% and yield % of different PNPs batches

**Phytomolecules**	**PNPs Batches No.**	**Yield %**	**Percentage entrapment efficiency (EE%)**	**%Drug loading**
RT	Batch 1-A	87%	76.32	69.38
	Batch 1-B	83.38%	62.22	56.56
	Batch 1-C	74%	73.15	66.5
QT	Batch 2-A	91.02%	73.28	66.62
	Batch 2-B	75%	67.85	61.68
	Batch 2-C	72.83%	57.38	52.16
TQ	Batch 3-A	81%	57.28	52.07
	Batch 3-B	75%	60.76	55.24
	Batch 3-C	70%	56.75	51.59

 It is well established that EE% and DL% are high when both polymer and phytomolecules have a high affinity to the same solvent. In this instance, RT, QT, TQ are poorly water soluble but possess high affinity to the same organic solvent in which the polymer was dissolved, thereby ensuring that there was no leakage of the hydrophobic phytomolecules to the aqueous phase during nanoparticles preparation resulted in high EE% and DL%.^32^ These pharmaceutical factors improved the entrapment of RT, QT, TQ into the polymer matrix.^33^

###  Particle size, polydispersibility index and zeta potential

 The particle size is considered as an important parameter as it affects EE%, release, solubility, absorption, bioavailability as well as the stability of the formulations.^34,35^ The particle sizes of RT-PNP, QU-PNP, TQ-PNP, were 412.4, 156 and 400 nm, respectively. This shows the developed PNPs were in the nanometric size range.

 The homogeneity of particle size distribution of batches 1-A, 2-A, 3-B of RT-PNP, QU-PNP, TQ-PNP were 0.35, 0.5 and 0.4, respectively, that was < 0.5, indicating size distribution within a narrow range. The zeta potential indicates the surface charge of the prepared PNPs and is an important factor for predicting the stability of the PNPs.^36^ The zeta potential of batches 1-A, 2-A, 3-B of RT-PNP, QU-PNP, TQ-PNP, were -18 mV, -26 mV, -56 mV, respectively. Higher negative zeta potential value is known to indicates good physical stability of the nanoparticles which stabilize the colloidal system as it overcomes the particle aggregation due to repulsive forces.^37^ Usually, the possibility of particle aggregation is much lower for charged particles with zeta potential > |20|.^38^ In the present study, the formulations of 1-A, 2-A, 3-B of RT-PNP, QU-PNP showed good physical stability.

###  Differential scanning calorimetry analysis

 Differential scanning calorimetry (DSC) is one of the most reliable technique to study compatibility, physico-chemical interactions between drug and excipient and assesses the physical state of the drug in the final developed formulation.^39,40^ The DSC thermograms clearly show the melting peak of blank formulation at 348.02 °C, RT at 175.10 °C, and 226.90 ˚C, RT-PNP at 165.09 °C and 289.06 °C, QT at 175.10 °C, QT-PNP at 383.56 °C and 385.81 °C, TQ at 97.33 °C and TQ-PNP at 351.20 °C and 418.37 °C.

 The sharp melting endothermic peaks of RT, QU, TQ were not detected in the respective thermogram of PNPs, indicating the absence of phytomolecules in a crystalline state. It may be concluded that, RT, QU, TQ were present in an amorphous state, following loading in Eudragit based polymeric nanoparticles, and may have dispersed homogeneously in the polymeric matrix.^41^

###  Transmission electron microscopy

**Figure 1:**
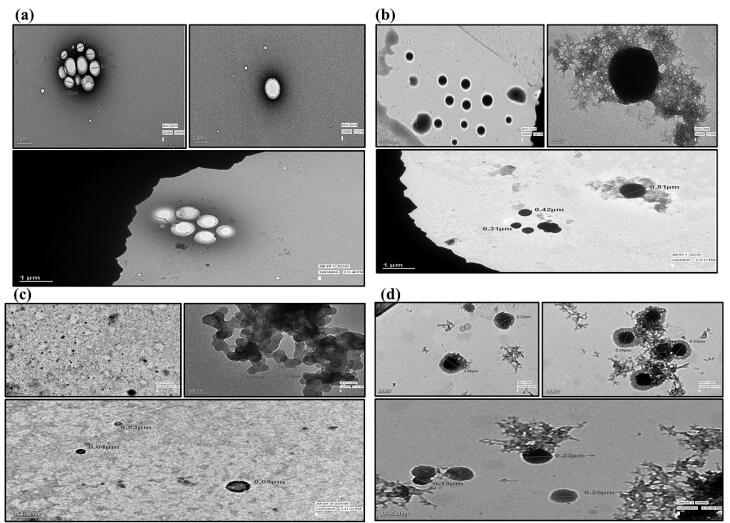
TEM images (Micrographs) **(a)** Blank formulation consist Eudragit polymer matrix, and appeared to be hollow without phytomolecules. **(b)** Morphology of RT-PNP, **(c)** QT-PNP and **(d)** TQ-PNP with nano scale

 The TEM images of RT-PNP, QU-PNP, TQ-PNP showed the presence of spherical PNPs with smooth surfaces and presence of well-dispersed nanoparticles sans aggregation, embedded within a polymeric matrix (Figure 1a-1d). On a scale of 1, 0.1, 0.2 µm, the average particle sizes of RT-PNP, QU-PNP, TQ-PNP were 446 ± 0.152, 39.6 ± 0.006 and 186 ± 0.513 nm, respectively (Figure 1a-1d). The larger particle size of RT-PNP may be attributed to the complex structure of the RT and availability of quaternary ammonium groups but low acrylic content of Eudragit RL 100 versus Eudragit RS 100. This causes an increase in the viscosity of the polymer organic phase solution which hinders its dispersibility into the aqueous phase, which may result in the formation of large size nanoparticles.^42^ The surfaces of RT-PNP, QU-PNP, TQ-PNP were of non-homogenous texture confirming that RT, QU, TQ were dispersed throughout the polymeric matrix.

###  Nuclear magnetic resonance

**Figure 2:**
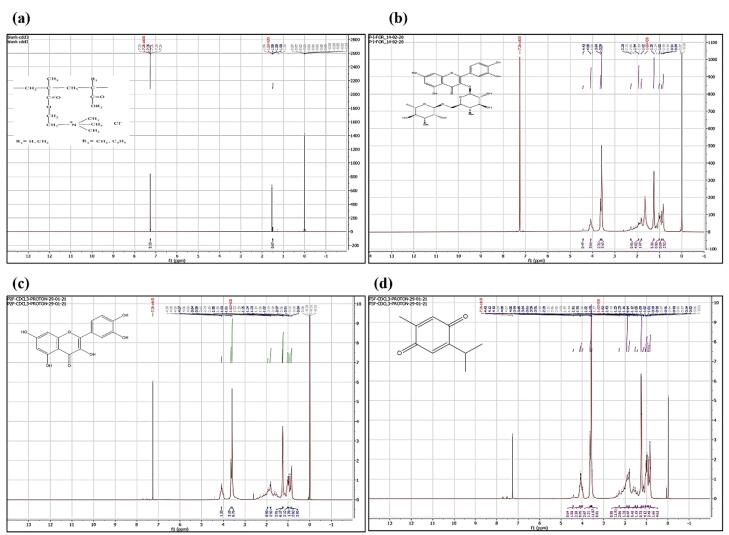
1H NMR spectra **(a)** Blank formulation (without phytomolecules), **(b)** RT-PNP, **(c)** QT-PNP, and **(d)** TQ-PNP

 The ^1^H NMR spectrum of a molecule provides the primary information about its structure, conformation, physical form (crystalline/amorphous) etc. The ^1^H NMR spectrum of blank-formulation, RT-PNP, QT-PNP, TQ-PNP showed characteristic peaks indicative of molecular dispersion of RT, QU, TQ in the polymeric matrix (Figure 2a-2d).

###  In vitro drug release

 The *in vitro *drug release was performed to evaluate the target release of RT, QU, TQ from RT-PNP, QU-PNP, TQ-PNP to the intestine. The batches 1-A, 2-A 3-B of RT-PNP, QU-PNP, TQ-PNP, respectively were selected for *in vitro *drug release study as they possessed the highest EE%, % yield as well as smallest particle size diameter.

**Figure 3:**
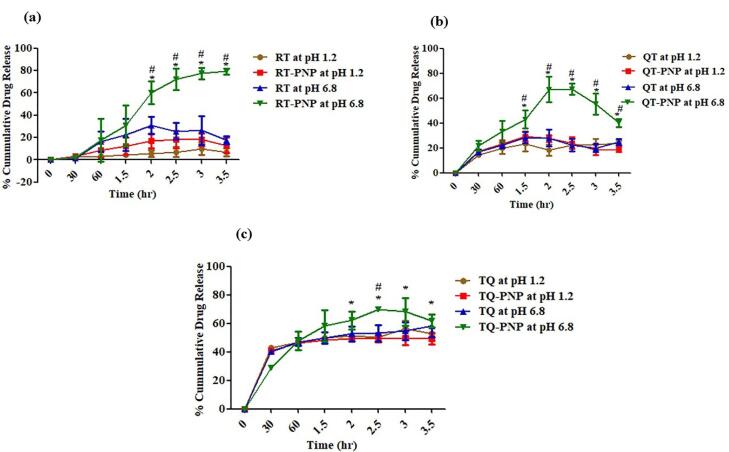
In-vitro release of **(a)** RT, **(b)** QT, **(c)** TQ; at stomach simulated fluid (pH 1.2) and intestinal simulated fluid at (pH 6.8)

 In simulated gastric fluid (pH 1.2), the dissolution of RT, RT-PNP, QT, QT-PNP, TQ and TQ-PNP upto 2 hours, were 5.544 ± 2.84, 17.08 ± 6.39, 18.55 ± 4.49, 27.6 ± 2.74, 51.34 ± 1.124 and 49.08 ± 1.35 %, respectively (Figure 3a-3c). In simulated intestinal fluid (pH 6.8), the dissolution of RT, RT-PNP, QT, QT-PNP, TQ and TQ-PNP upto hours, were 30.8 ± 7.75, 60.37 ± 10.08, 27.6 ± 2.7, 66.9 ± 10.31, 49.08 ± 1.35 and 62.09 ± 6.46 %, respectively (Figure 3a-3c).

 After 2 hr, the rate of releases of RT, RT-PNP, QT, QT-PNP, TQ, TQ-PNP and RT-PNP were 19.5 ± 0.54, 45.07 ± 0.89, 13.91 ± 4.69, 45.4 ± 6.72, 27.71 ± 3.27 and 32.95 ± 0.89, respectively which was twofold higher at intestinal fluids (pH 6.8) (Table 3). The batches 1-A, 2-A and 3-B of RT-PNP, QU-PNP, TQ-PNP, showed initial burst release upto 30 minutes followed by gradual release of RT, QU, TQ, maximum upto 2.5 hours, respectively, from the Eudragit polymer matrix at intestinal pH.

**Table 3:** The % of phytomolecules release (*in vitro* release profile)

**Time (h)**	**RT**	**QT**	**TQ**	**RT-PNP**	**QT-PNP**	**TQ-PNP**
0.5	1.13 ± 0.73	16.87 ± 2.47	40.38 ± 0.96	2.0551.079	21.73 ± 4.10	28.66 ± 1.18
1	15.19 ± 8.48	5.6 ± 0.3	6.51 ± 2.68	15.57 ± 8.06	11.5 ± 4.38	19.32 ± 5.33
1.5	11.7 ± 0.24	13.36 ± 3.0	18.48 ± 2.29	18.83 ± 5.62	20.87 ± 1.51	26.28 ± 6.79
2	19.5 ± 0.54	13.91 ± 4.69	27.71 ± 3.27	45.07 ± 0.89	45.40 ± 6.72	32.95 ± 0.89
2.5	13.33 ± 4.23	11.02 ± 2.21	32.23 ± 3.62	48.27 ± 5.62	40.69 ± 0.37	44.95 ± 2.46
3	17.91 ± 10.5	12.57 ± 1.62	36.86 ± 4.28	53.28 ± 1.98	32.74 ± 7.07	44.89 ± 9.77
3.5	11.08 ± 0.11	19.08 ± 1.46	42.63 ± 6.26	57.2 ± 1.16	24.31 ± 0.70	42.24 ± 1.65

 The polymer ratio Eudragit RS100: RL100:: 30:70, exhibited controlled release of phytomolecules, as they can swell yet remain insoluble at physiological pH values and have strong electrostatic contact, which affects the release.^43^ Eudragit RS can be attributed to the low permeability of the polymer, which posed a significant hindrance to fluid penetration and passive drug diffusion and the release of phytomolecules from the unique polymer matrix may be attributed to ammonium groups present in salt form and makes the polymers porous with slow swelling of the matrix at intestinal pH 6.8, followed by dissolution of the phytomolecule in the intestinal fluid that was not seen at gastric pH.^44^ This confirms that the unique ratio of Eudragit polymers is an effective matrix for intestinal drug release (Figure 3).

 The release of RT, QU, TQ from RT-PNP, QU-PNP, TQ-PNP increased at higher pH i.e., intestinal simulated media, and achieved the objective of the formulation.

###  Drug release kinetics

**Figure 4:**
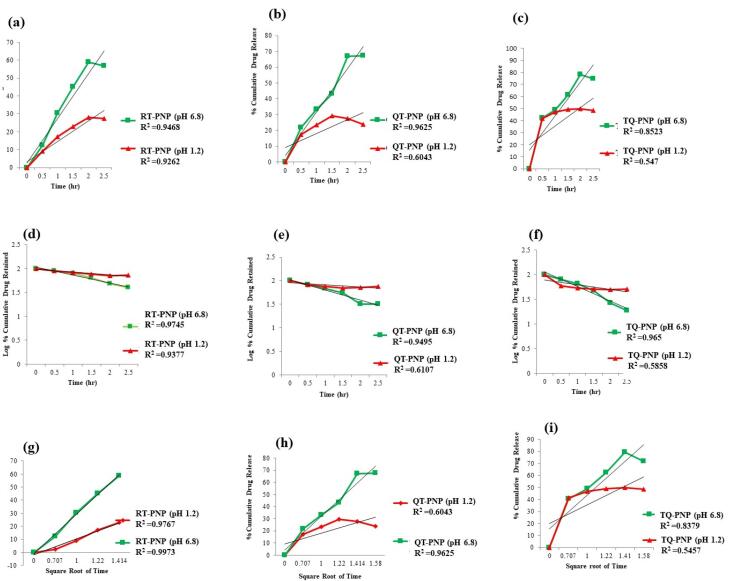
In-vitro release kinetics **(a)** zero order release of RT-PNP, **(b)** QT-PNP, **(c)** TQ-PNP.** (d)** First order release of RT-PNP **(e) **QT-PNP, **(f)** TQ-PNP. **(g)** Higuchi release of rutin-PNPs, **(h) **QT-PNP, **(i) **TQ-PNP

 Mathematical modelling of the release profiles of phytomolecule with different kinetic equations and the regression coefficients (*R*^2^) for RT-PNP, QU-PNP, and TQ-PNP were calculated (Table 3). The optimized batches 1-A, 2-A, 3-B of RT-PNP, QU-PNP, TQ-PNP showed uniform release in terms of their correlation coefficients for Higuchi diffusion controlled mode as 0.972, 0.980, 0.929, respectively (Table 4; Figure 4a-4i). The Higuchi order release kinetic model exhibited highest R^2^ value for RT-PNP, QU-PNP, and TQ-PNP as compared to other applied kinetics model. The ‘n’ values of RT-PNP, QU-PNP, and TQ-PNP were 0.337, 0.419, 0.33, respectively, which demonstrated that release of RT, QU, TQ from the developed PNPs, was through Fickian diffusion^45^ (Figure 4a-4i).

**Table 4:** Best fit model for PNPs-formulation batch

**Formulation**	**Zero order**	**First order**	**Higuchi matrix**	**Peppas plot**	**N**	**Best fit model**
	**(r**^2^**)**	**(r**^2^**)**	**(r**^2^**)**	**(r**^2^**)**		
RT-PNPs (pH 1.2)	0.392	0.788	0.875	0.592	0.177	Higuchi
RT-PNPs (pH 6.8)	0.929	0.831	0.972	0.831	0.337	Higuchi
QT-PNPs (pH 1.2)	0.936	0.764	0.980	0.764	0.419	Higuchi
QT-PNPs (pH 6.8)	0.936	0.764	0.980	0.764	0.419	Higuchi
TQ-PNPs (pH 1.2)	0.654	0.542	0.654	0.555	0.185	Zero
TQ-PNPs (pH 6.8)	0.902	0.703	0.905	0.666	0.257	Higuchi

N: Higuchi mechanism slope equation line.

 Based on the results, it may be proposed that the mechanism of release of RT, QU, TQ from the Eudragit polymer matrix may be combination of slow swelling (polymer relaxation) and gradual erosion (polymer dissolution) at intestinal but not gastric pH.^46,47^

###  Stability study

**Figure 5:**
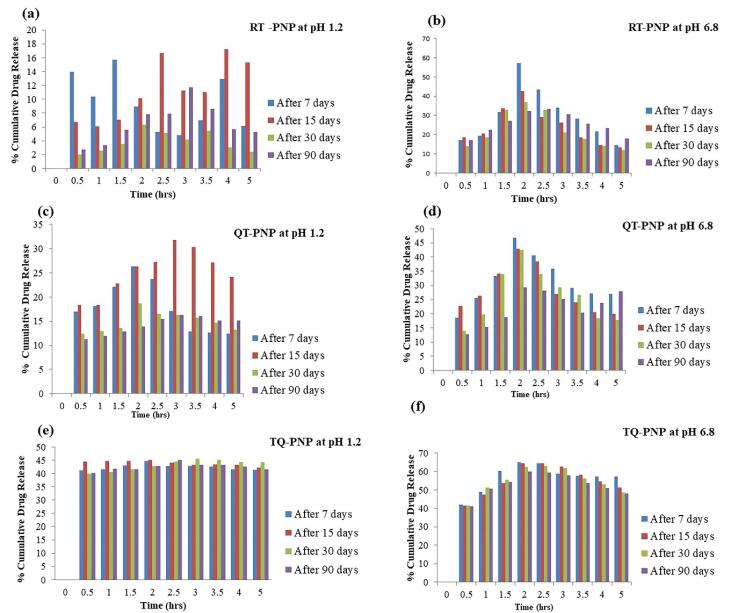
In-vitro release of RT, QU, TQ from stored RT-PNP, QU-PNP, TQ-PNP at stomach simulated fluid (pH 1.2) and intestinal simulated fluid at (pH 6.8) **(a-f)**

 Optimized batches (1-A, 2-A, 3-B) of RT-PNP, QU-PNP, TQ-PNP, were subjected to stability study and compared against freshly prepared batches for EE%, DL% and *in vitro* release of RT, QU, TQ (Table 5; Figure 5a-5f).

**Table 5:** EE% and DL% of optimized PNPs batches after storage condition

**Phytoconstituents loaded nanoparticles (PNPs)**	**Days**	**Percentage entrapment efficiency (EE%)**	**Drug loading (%)**
RT-PNPs	7	60.38	54.89
	15	58.98	53.62
	30	56.78	51.62
	90	45.28	41.16
QT-PNPs	7	85.45	77.68
	15	73.28	66.61
	30	61.85	56.23
	90	52.20	47.45
TQ-PNPs	7	70.25	63.86
	15	59.52	54.11
	30	56.73	51.57
	90	45.87	41.70

 After 3 months of storage, neither aggregation nor irregularity was observed, that may be due to the presence of stabilizer.^48^ The EE%, DL% and *in vitro* release of RT, QU, and TQ from stored and freshly prepared RT-PNP, QU-PNP, and TQ-PNP were not significantly different.

###  Ex-vivo intestinal permeability 

**Figure 6:**
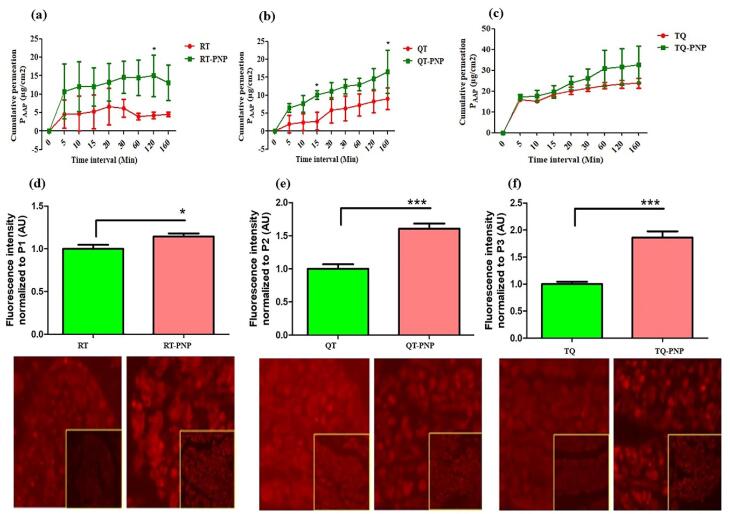
Aapparent permeation coefficient (P_APP_) of RT, QU, TQ, RT-PNP, QU-PNP, TQ-PNP across the small intestine **(a-c). **Data expressed as mean ± SD; (n = 6), *p < 0.05 vs RT, QU, TQ. The depth of permeation of RT-PNP, QU-PNP, TQ-PNP across intestinal epithelium studied by CLSM **(d-f).** Data expressed as mean ± SD; (n = 6), *P < 0.5 and ***P < 0.001 vs RT, QU, TQ

 The apparent permeation coefficients of RT, QU, TQ, RT-PNP, QU-PNP, TQ-PNP across the small intestine at 160 minutes, were 4.52 ± 0.612, 9.07 ± 3.04, 23.84 ± 2.4, 13.1 ± 4.82, 16.6 ± 6.01, 32.78 ± 8.88 µg/cm^2^, respectively (Figure 6a-6c). The intestinal permeation of RT-PNP, QU-PNP, TQ-PNP were significantly (*P* < 0.05) higher than RT, QU, TQ (Figure 6). The higher intestinal permeability has been largely attributed to the small size of PNPs which increases contact surface area and prolongs intestinal drug residence time. In addition, Eudragit RL100 and Eudragit RS100 bypass the gastric pH and permeable in digestive fluids and positively charged facilitates muco-adhesive to the intestinal epithelial layer and deep penetration into the intervillous space leading to a higher diffusion rate of the drug.^49,50^ In addition, reduction in particle size can cause augmented dissolution and saturation solubility which increases the concentration gradient between the intestinal epithelial cells and the underlying mesenteric circulation resulting in improved phytomolecules absorption.^51^ PVA as surfactant used in the formulation could also have contributed to the enhanced permeability because they are surface-active agents capable of altering membrane fluidity leading to improved drug absorption across the gut.^52^ The CLSM was used to visualize and further confirm the intestinal permeability of the RT-PNP, QU-PNP, and TQ-PNP (Figure 6d-6f). The intestinal tissue was observed along the ‘*z*’ axis to evaluate the depth of fluorescence permeated through the layers of intestinal section. The depth of permeation of RT-PNP, QU-PNP, TQ-PNP were increased significantly (*P* < 0.05) (Figure 6). The deeper permeation of fluorescence might be the direct consequence of PNPs as well as modulation of intestinal epithelium by polymer used in nanoparticle formulation. The results from the CLSM study further indicate that the intestinal permeation of RT, QU, and TQ was improved from developed PNPs.

## Conclusion

 In this study, we prepared Eudragit based freeze dried nanoparticles (PNPs) encapsulated in a capsule as a final formulation system. The high % yield and EE% can be attributed to the optimized percentage of Eudragit RS100:RL 100::30:70, depending on its quaternary ammonium salt. The optimized PNPs showed good physiochemical stabilities and exhibited maximal releases of phytomolecules in the simulated intestinal fluid. The spherical sizes obtained in nanometric range were responsible for their high permeation across the intestinal epithelium. The developed PNPs appear to be a good approach to increase the permeability of the hydrophobic phytomolecules such as RT, QT and TQ.

## Competing Interests

 The authors report there are no competing interests to declare.

## Data Availability Statement

 The authors confirm that the data supporting the findings of this study are available within the article

## Ethical Approval

 The protocol of the animal study was approved by the Institutional Animal Ethics Committee DPSRU (IAEC 2019/II 09).
